# Facilitating full and effective implementation of the Paris Agreement for carbon neutrality vision

**DOI:** 10.1007/s43979-022-00014-8

**Published:** 2022-04-19

**Authors:** Huaqing XU

**Affiliations:** National Center for Climate Change Strategy and International Cooperation, Beijing, 100035 China

## Abstract

The Paris Agreement, a landmark in the multilateral process of global climate governance, not only demonstrates the greatest inclusiveness and feasibility based on science and principles, but also points out the general direction of the global green and low-carbon transition. The Agreement has set a global goal to hold the increase in the global average temperature to well below 2 degrees Celsius above pre-industrial levels, and to pursue efforts to limit the temperature increase to 1.5 degrees Celsius by the end of the century. To achieve this long-term objective, developed countries should take the lead in reducing emissions as soon as possible, which is fundamental to the achievement of net-zero global emissions at an early date. China’s goal of striving to peak carbon dioxide emissions before 2030 and achieve carbon neutrality before 2060 shows its great ambition, strength, and responsibility as a major country, indicating that China is committed to realizing carbon neutrality from carbon peaking in the shortest time in global history, and will make greater efforts and contributions to achieve the goals set out in the Paris Agreement.

Chinese President Xi Jinping delivered an important statement at the General Debate of the 75th Session of the United Nations General Assembly on 22 September 2020, announcing that China would aim to peak carbon dioxide emissions before 2030 and achieve carbon neutrality before 2060 [1]. This is a solemn commitment made by President Xi to the international community for realizing the Chinese Dream of national rejuvenation and for adjusting to the unprecedented changes in the world. It is also a major strategic decision made by the Central Committee of the Communist Party of China (CPC) with Xi Jinping at its core in light of overall domestic and international situations, to build a community with a shared future for mankind, and meet the inherent requirement of achieving sustainable development of the Chinese nation.

At the 76th Session of the United Nations General Assembly on 21 September 2021, President Xi reaffirmed China’s commitment to striving to peak carbon dioxide emissions before 2030 and achieve carbon neutrality before 2060, noting that China would do its utmost despite all the hard work [2]. China will step up support for other developing countries in developing green and low-carbon energy and will not build new coal-fired power projects abroad as shown in Fig. 1. This further demonstrates the confidence, courage, and responsibility of leaders of responsible major developing countries.

**Fig. 1:**
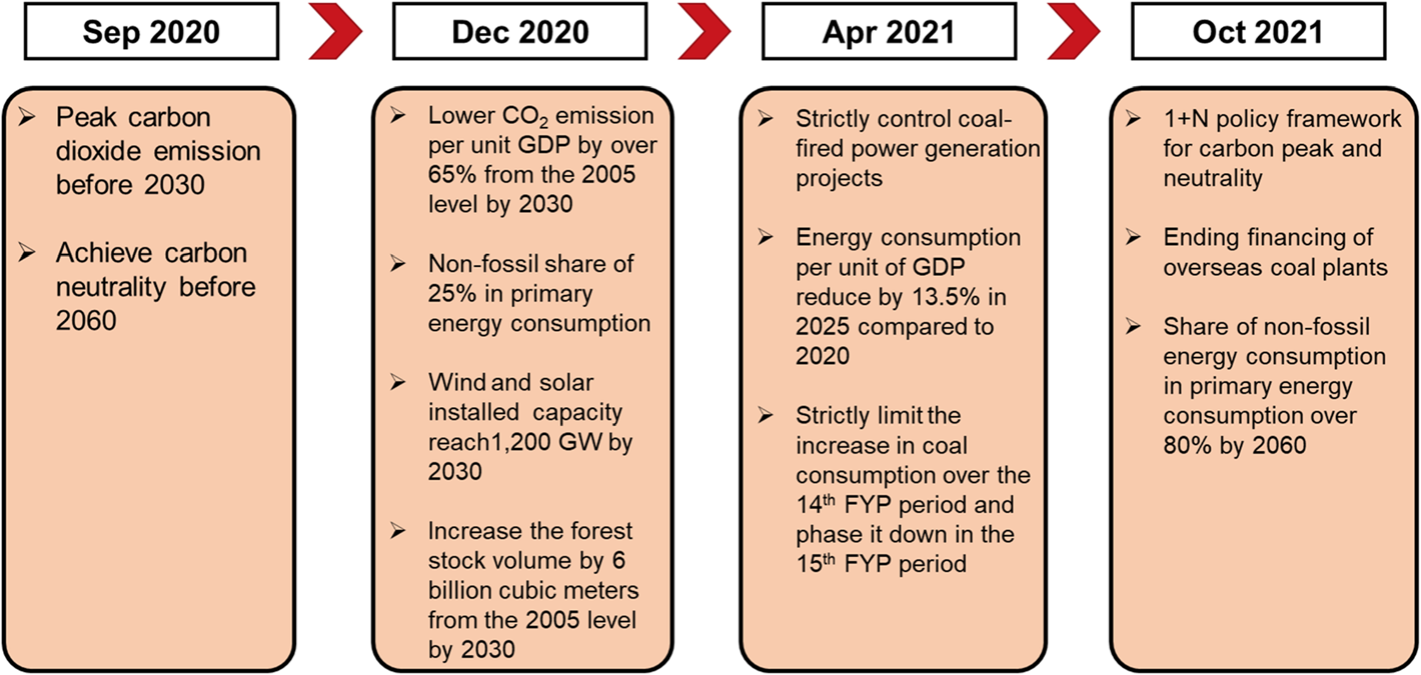
China’s major climate goals

**China’s important announcement of the carbon neutrality vision has greatly boosted the international community’s ambition and confidence in the effective implementation of the Paris Agreement.** President Xi noted that the Paris Agreement [3] charts the course for the world to transition to green and low-carbon development, and requests that all countries must take decisive steps as this is the minimal requirement for an all-nation effort to protect the Earth. The announcement has released a positive signal to the international community that China will firmly follow the path of green and low-carbon development and lead the construction of a global ecological civilization and a beautiful world, boosting the world’s ambition and confidence, and contributing the Chinese wisdom and solutions to the global climate security issues faced by the human community. The announcement has greatly promoted the implementation of the objective of achieving “a balance between anthropogenic emissions by sources and removals by sinks of GHGs in the second half of the century” outlined in the Paris Agreement, bridged the gap in global emissions and injected strong impetus for the international community to fully and effectively implement the Paris Agreement. Furthermore, it demonstrates China’s responsibility and commitment to taking a driving seat in international cooperation on climate change and working toward a fair and equitable system of global climate governance based on mutually beneficial cooperation, and promoting the building of a shared future for mankind as an important participant, contributor and torchbearer in the global endeavor for ecological civilization.

**The important announcement of China’s carbon neutrality vision highlights the direction and goal of China’s green, low-carbon and high-quality development.** According to President Xi, the COVID-19 pandemic has revealed that a self-revolution for mankind is needed to accelerate the formation of green production modes and living patterns and to build an ecological civilization and a beautiful planet. China’s carbon neutrality vision depicts a blueprint for achieving green, low-carbon and high-quality development in the future, which is highly consistent with its goal of building a modern and powerful country. It is a must, in starting a new journey of building a modern socialist country, to seize the historic opportunity of a new round of technological revolution and industrial change under the target of carbon neutrality. The announcement not only determines the direction to firmly follow the green and low-carbon high-quality development path that prioritizes ecological civilization, comprehensively build a powerful modern socialist country and realize the sustainable development of China, but also sets out objectives for China to promote innovation in green and low-carbon science and technology, and accelerate the formation of new green and low-carbon economic dynamics and sustainable growth.

Recently, China released two directives titled “Working Guidance for Carbon Dioxide Peaking and Carbon Neutrality in Full and Faithful Implementation of the New Development Philosophy” [4] and the “Action Plan for Carbon Dioxide Peaking before 2030 [5].” Specific implementation plans for key areas including energy, industry, construction and transport, and for key sectors such as coal, electricity, iron and steel, and cement will be rolled out, coupled with supporting measures in terms of science and technology, carbon sink, fiscal and taxation, and financial incentives, etc. With these measures, China aims to form a “1 + N” policy framework for achieving carbon peak and carbon neutrality, with clearly defined timetable, roadmap and blueprint. To do a good job in the research and support of carbon peak and carbon neutrality, we need to focus on the following aspects.

**First, adhering to the guidance of the new development philosophy.** It is necessary to fully, accurately and comprehensively implement the new development philosophy, firmly implement the national strategy of actively addressing climate change, step up efforts to tackle climate change, and stick to the green and low-carbon high-quality development path. It is essential to synergize the reduction of pollution and carbon emissions, which is key to accelerating the green transformation of economic and social development. By effectively controlling the emissions of carbon dioxide while improving the climate governance system, the goal of peaking carbon dioxide emissions before 2030 will be better achieved. We should strike a balance between the economic and social development, protecting energy security and addressing climate change, coordinate pollution control and ecological protection while tackling climate change, and resolutely curb the irrational development of energy-intensive and high-emission projects.

**Secondly, concentrating on the leading and backward role of the two major goals of peaking carbon dioxide emissions and reaching carbon neutrality.** We need to be determined to the national strategy, strengthen the national strategic intent, and scientifically understand and accurately grasp the strategic direction of the two “before”, set targets and take actions with scientific cognition as shown in Fig. 2. We should be fully devoted to achieving the carbon neutrality goal before 2060, continuously enhancing rigid constraints of the target of greenhouse gas emissions control, and creating conditions to shift from controlling energy consumptions to controlling the amount and intensity of carbon emissions as soon as possible. We should follow the objectives of “striving to build a modernized and harmonious coexistence between human beings and nature”, conduct research and formulate a carbon neutrality roadmap, accelerate the green and low-carbon structural transformation and technological innovation, and avoid taking the old road of the traditional terminal treatment of pollutants.

**Fig. 2:**
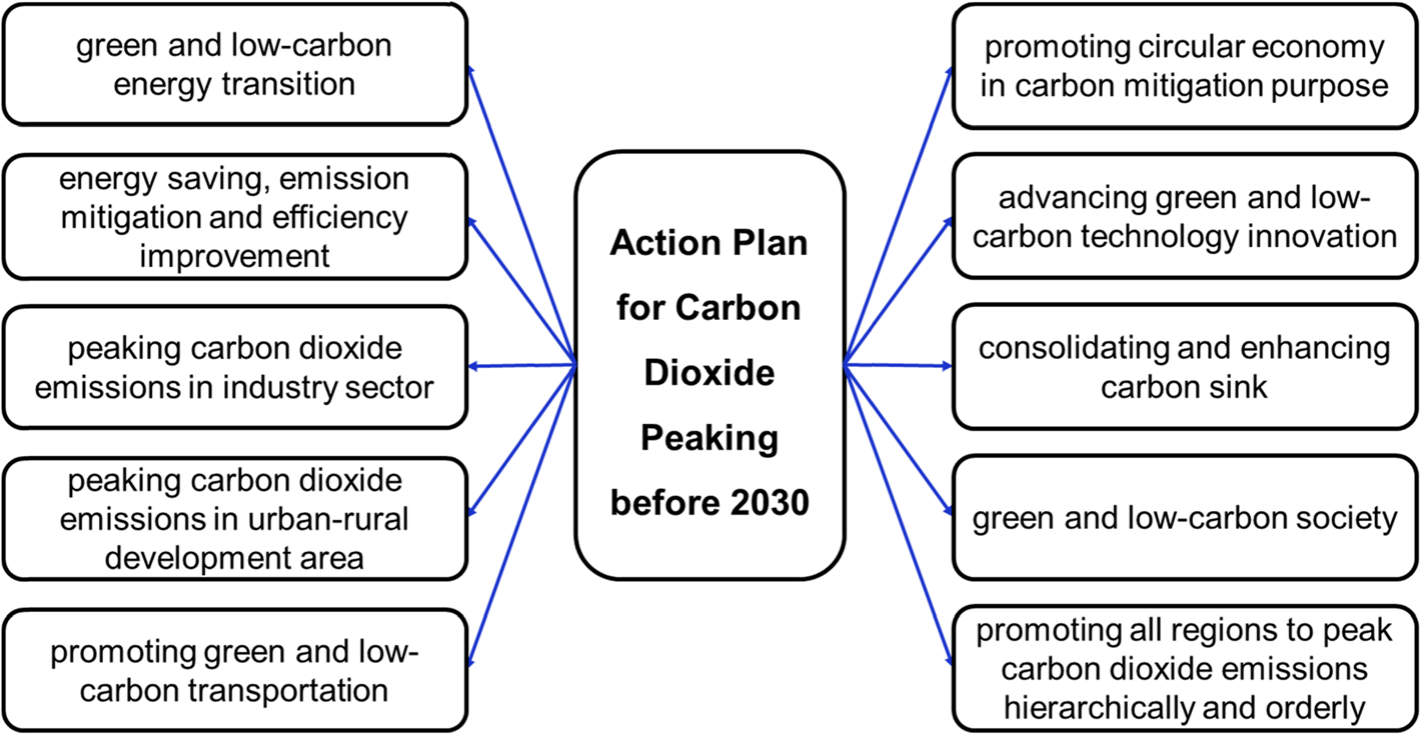
Action plan for carbon dioxide peaking before 2030

**Thirdly, supporting regions with favorable conditions, key industries and key enterprises in taking the lead in peaking carbon dioxide.** China will proceed the work on peaking carbon emissions in a vigorous, orderly and effective manner, support regions with optimal conditions to take the lead in peaking carbon emissions before 2020, strive to peak carbon emissions in heavy chemical industries around 2020, support areas with favorable conditions to take the lead in reaching peak carbon emissions, and strive to peak carbon emissions in the industrial sector as soon as possible. Central enterprises should formulate implementation plans for the peaking goal.

**Last but not the least, accelerating breakthroughs in the four major revolutions, namely industrial revolution, energy revolution, technological revolution and consumption revolution.** The industrial revolution should integrate the development of digitalization, intellectualization and decarbonization, while focusing on the promotion of green manufacturing. The energy revolution should prioritize energy resource conservation, highlight the low-carbon development of energy, and strictly control coal power projects and coal consumption. The technological revolution should strengthen the national power of strategic science and technology, and achieve breakthroughs in low carbon frontier research, based on the development of novel energy technologies, new energy vehicles, low-emission infrastructure and green, zero-carbon industries. The consumption revolution should advocate simplified and moderate consumption patterns, green and low-carbon travel modes, and waste sorting habits, promote social civilization, actively create a new green and low-carbon living style, and strive to build a modernized and harmonious coexistence between mankind and nature.

As the Chinese saying goes, “successful governance relies on solid actions.” President Xi made three suggestions on tackling climate change in a written statement at the COP26 World Leaders Summit. First, upholding multilateral consensus, as multilateralism is the right prescription when it comes to global challenges such as climate change. Second, focusing on concrete action, as only actions can turn visions into reality. Third, accelerating the green transition. It is significant to harness scientific innovations to transform and upgrade energy resources, industrial structure, and consumption pattern, to promote green economic and social development, and explore a new pathway forward that coordinates development with conservation. China also calls on all parties to take stronger actions to jointly address the climate challenge and work together to protect the earth, a shared home for all mankind.
